# Bacterial interactome disturbance in chronic obstructive pulmonary disease clinical stability and exacerbations

**DOI:** 10.1186/s12931-024-02802-5

**Published:** 2024-04-20

**Authors:** Wei Xiao, Yi-long Chen, Long-yi Du, Jiqiu Wu, Zhang Wang, Bing Mao, Fu-qiang Wen, Peter Gerard Gibson, Vanessa M. McDonald, Haopeng Yu, Juan-juan Fu

**Affiliations:** 1 Division of Pulmonary Medicine, Department of Internal Medicine, Institute of Integrated Traditional Chinese and Western Medicine, West China Hospital of Sichuan University, No. 37, Guoxue Lane, Wuhou District, Chengdu, 610041 China; 2 Divison of Pulmonary diseases, State Key Laboratory of Biotherapy, West China Hospital of Sichuan University, Chengdu, China; 3 West China Biomedical Big Data Center, West China Hospital of Sichuan University, Chengdu, China; 4 Med-X Center for Informatics, Sichuan University, Chengdu, China; 5 Department of Genetics, University Medical Center Groningen, University of Groningen, Groningen, The Netherlands; 6 Institute of Ecological Sciences, School of Life Sciences, South China Normal University, Guangzhou, China; 7 Department of Respiratory and Critical Care Medicine, West China Hospital of Sichuan University, Chengdu, China; 8 Priority Research Centre for Healthy Lungs, The University of Newcastle, Newcastle, NSW Australia

**Keywords:** Chronic obstructive pulmonary disease, Exacerbation, Airway dysbiosis, Microbial interactome, Antagonistic interaction

## Abstract

**Rationale:**

Our understanding of airway dysbiosis in chronic obstructive pulmonary disease (COPD) remains incomplete, which may be improved by unraveling the complexity in microbial interactome.

**Objectives:**

To characterize reproducible features of airway bacterial interactome in COPD at clinical stability and during exacerbation, and evaluate their associations with disease phenotypes.

**Methods:**

We performed weighted ensemble-based co-occurrence network analysis of 1742 sputum microbiomes from published and new microbiome datasets, comprising two case-control studies of stable COPD versus healthy control, two studies of COPD stability versus exacerbation, and one study with exacerbation-recovery time series data.

**Results:**

Patients with COPD had reproducibly lower degree of negative bacterial interactions, i.e. total number of negative interactions as a proportion of total interactions, in their airway microbiome compared with healthy controls. Evaluation of the *Haemophilus* interactome showed that the antagonistic interaction networks of this established pathogen rather than its abundance consistently changed in COPD. Interactome dynamic analysis revealed reproducibly reduced antagonistic interactions but not diversity loss during COPD exacerbation, which recovered after treatment. In phenotypic analysis, unsupervised network clustering showed that loss of antagonistic interactions was associated with worse clinical symptoms (dyspnea), poorer lung function, exaggerated neutrophilic inflammation, and higher exacerbation risk. Furthermore, the frequent exacerbators (≥ 2 exacerbations per year) had significantly reduced antagonistic bacterial interactions while exhibiting subtle compositional changes in their airway microbiota.

**Conclusions:**

Bacterial interactome disturbance characterized by reduced antagonistic interactions, rather than change in pathogen abundance or diversity, is a reproducible feature of airway dysbiosis in COPD clinical stability and exacerbations, which suggests that we may target interactome rather than pathogen alone for disease treatment.

**Supplementary Information:**

The online version contains supplementary material available at 10.1186/s12931-024-02802-5.

## Introduction

Chronic obstructive pulmonary disease (COPD) is a worldwide health challenge, ranking third leading cause of death globally [1]. It is featured by persistent airway inflammation, emphysema, and airflow obstruction, with episodes of exacerbations accelerating disease progress [1].

Increasing evidence suggest that airway dysbiosis contributes to COPD: alterations of human airway microbiome are correlated with lung immunology, exacerbation frequency, and mortality [2–4]; and depletion of murine microbiota mitigates chronic pulmonary inflammation [5]. However, the design of interventions to restore microbiota remains difficult due to lack of a clear picture of COPD dysbiosis. Microbial features including diversity metrics and differentially abundant taxa identified in previous microbiome studies are very inconsistent, either at COPD clinical stability or during exacerbation (Supplementary Tables 1–2). Furthermore, although alterations in alpha diversity and relative abundance of taxa within the *Proteobacteria*, *Firmicutes*, and *Bacteroidetes* are reported in COPD stability, there is minor change in airway microbiome during exacerbation [6]. These ambiguities urge the need to understand airway dysbiosis in an alternative prospective.

Human-associated microbiomes are ecosystems where microbes do not exist in isolate but form complex interaction networks, known as the interactome [7]. Unravelling the interactions between gut microbiota and pathogenic species has promoted the clinical success of fecal microbiota transplantation for the treatment of *Clostridium difficile* [8] and inflammatory bowel diseases [9]. Compared to intestinal microbiota, the airway microbiota is a low-biomass organ, yet in which different bacterial species can still exert potent interactions: airway derived *Rothia mucilaginosa* mitigates *Pseudomonas aeruginosa* induced inflammation in a 3-D alveolar epithelial model [10]; and episodic aspiration of oral commensals effectively decrease *Streptococcus pneumonia* susceptibility in mice [11]. However, the role of these microbial interactions in COPD has not been considered in previous microbiome studies that exclusively focused on differential abundance or diversity metrics.

Here we present a large-scale network analysis of sputum microbiomes (*n* = 1,742) from 5 geographically diverse cohorts including cross-sectional and longitudinal data to explore the role of airway bacterial interactome in COPD. We demonstrate that bacterial interactome is disturbed at both COPD stability and exacerbation and strongly associated with exacerbation phenotype, which expand our current knowledge regarding COPD dysbiosis and shed light on future therapeutic design.

## Methods

### A prospective chinese cohort of COPD patients and controls

The EndAECOPD (Endotype-Driven Prediction of Acute Exacerbations in COPD) cohort aimed to identify biological predispositions to exacerbations of COPD in Chinese populations with study protocol published previously [12]. Between May, 2018 and Jan, 2020, 165 stable COPD patients free of antibiotics in the past two months were consecutively enrolled and underwent sputum induction at the West China Hospital. In total, 143 patients provided a qualified sputum sample with sufficient DNA for 16S rRNA gene sequencing. Patients were followed by telephone interview every three months for 1-year to monitor their exacerbations. An additional 44 healthy volunteers recruited at the same center were sequenced later as part of this work.

### Sputum induction and sample assays

Sputum induction with stringent oral cleaning to decrease contamination was performed according to standard protocols [12]. Sputum plug was aliquoted for 16S rRNA gene analysis, bacterial DNA quantification, inflammatory cell counting, and cytokine detection. After bacterial DNA isolation, the V3-V4 region of the 16S rRNA gene was amplified and sequenced on the Illumina MiSeq PE300 platform [12] with proper reagent controls (Supplementary Methods and Supplementary Fig. 1). The raw sequence data have been deposited in the Genome Sequence Archive in National Genomics Data Center (HRA003966). Total bacterial DNA was quantified by using an ABI7,500 qPCR system (Applied Biosystems, USA) with the PCR primers 5′- ACTCCTACGGGAGGCAGCAG-3′ and 5′- ATTACCGCGGCTGCTGG-3′. A panel of 19 sputum inflammatory mediators including interleukin (IL) − 1β, IL-2, IL-4, IL-5, IL-6, IL-8, IL-10, IL-12p70, IL-13, IL-17, IL-18, IL-23, IL-33, interferon-γ, C-X-C motif chemokine ligand 1, granulocyte-macrophage colony-stimulating factor, S100A8, S100A9 and matrix metalloproteinase-12 was assayed by using a Human Magnetic Luminex Assay kit (R&D Systems, USA).

### Public sputum microbiome datasets

We retrieved public 16S rRNA gene sequencing data of sputum samples from patients with COPD at either clinical stability or exacerbation and healthy controls (HC) by exhausted literature search in PubMed and dataset search in the Short Read Archive and the European Nucleotide Archive databases. The search term and inclusion/exclusion criteria are available in Supplementary Table 3. Only case-control studies of stable COPD *versus* HC or studies comparing COPD stability *versus* exacerbation were included. Studies that recruited subjects with respiratory diseases other than COPD, had relevant information indicating antibiotic use in the past month prior subject enrollment, or had sample size of < 20 for individual group (HC or COPD) after data processing (illustrated below), were excluded.

### Microbiome data preprocessing and taxonomic profiling

All 16S rRNA gene datasets were processed using a standardized pipeline in QIIME 2.0 (Quantitative Insights Into Microbial Ecology) [13]. The demultiplexed sequencing reads were merged, denoised, and resolved into amplicon sequence variants (ASVs) using DADA2 (Divisive Amplicon Denoising Algorithm 2) [14]. Samples with < 1,000 clean reads were discarded. Taxonomy assignment was performed with a custom Naive Bayes classifier trained on Greengenes Database 13_8 99% operational taxonomic units. Alpha-diversity (Shannon index) and beta-diversity (weighted UniFrac distance) metrics were calculated. Principal coordinates analysis was performed and permutational multivariate analysis of variance (999 permutations) was used for significance testing. Taxa with a prevalence of > 5% and an abundance of > 0.05% were kept for further analysis. Linear discriminant analysis effect size (LEfSe) analysis was used to identify differentially abundant taxa [15].

### Microbial co-occurrence network analysis

To identify microbial association networks while suppressing spurious correlations due to compositional effects of microbiome data, we implemented the weighted ensemble-based co-occurrence analysis along with Reboot [16, 17]. This approach calculated five correlation measures including Mutual Information, Bray-Curtis dissimilarity, Spearman and Pearson correlation, and generalized boosted linear models (GBLM), and merged the edge *P* values from the ensemble networks using the weighted Simes test. Then the network edge scores were merged as a weighted aggregate of the normalized absolute edge scores and the sign assigned based on GBLM, Spearman, and Pearson correlation (more details described at https://github.com/translational-respiratory-lab/The_Interactome). Co-occurrence networks were visualized in Cytoscape 3.9 and network parameters including node degree (the number of edges connected to a particular node), betweenness centrality (the fraction of shortest paths passing through a node), and stress centrality (the absolute number of shortest paths passing through a node) were calculated [18].

### Microbial network clustering and prediction of exacerbations

Spectral clustering was performed on the combined patient network to identify homogeneous COPD subtypes [17, 19]. The optimal number of clusters was determined by using the eigengap method, with a bootstrapping approach implemented to assess the robustness of identified clusters [17]. A life-table analysis was used to evaluate the ability of network clusters in prediction of time to next exacerbation.

## Results

### Consistent processing of published and new microbiome datasets included in this study

To identify reproducible associations between airway microbiota and COPD, we performed 16S rRNA gene sequencing of sputum samples of 187 patients with COPD and HC recruited from the EndAECOPD cohort (see Supplementary Table 4 for subject characteristics), and analyzed them in the context of 1,555 additional sputum samples from four publicly available and geographically diverse datasets. In total, two case-control studies of stable COPD versus HC, two studies of COPD stability versus exacerbation, and one study with exacerbation-recovery time series data were included (Table 1 [6, 20–22]). All of these datasets had considerable sample size. Except for SRP066375 [20], other datasets including COPDMAP [6], BCCS & BCES [21], and BEAT-COPD [22] were published with detailed study design and subject demographics. Although unpublished, SRP066375 showed comparable quality control statistics with published datasets (Supplementary Table 5), indicative of its reasonable data quality for downstream analysis.

**Table 1:** Characteristics of the large-scale COPD microbiome datasets included in this study

Analysis plan	Study/Dataset	Group	No. of samples	Sample types	Country	Primer	Platform	Reads per sample‡ Median (IQR)
Health-COPD comparative analysis	EndAECOPD (This study)	HC	44	IS	China	V3-4	MiSeq	48,726 (40,853–55,601)
		COPD	143					
	SRP066375 [20]	HC	116	IS	China (50), Nepal (50) Bangladesh (49), Peru (41)	V4	MiSeq	34,890 (28,377–40,453)
		COPD	74					
Stability-exacerbation comparative analysis	COPDMAP [6]	Stability	445	IS, SS	UK	V4	MiSeq	50,941 (46,905–56,712)
		Exacerbation	270					
	BCCS & BCES [21]	Stability	80	IS, SS	Norway	V3-4	MiSeq	26,777 (17,709–34,457)
		Exacerbation	94					
Exacerbation-recovery time series analysis	BEAT-COPD [22]	Stability	106	IS, SS	UK	V3-5	454	9,304 (7,472–11,514)
		Exacerbation	137					
		Post-therapy*	136					
		Recovery†	97					

Definition of abbreviations: BCCS & BCES = Bergen COPD cohort study & Bergen COPD Exacerbation Study, BEAT-COPD = Biomarkers to Target Antibiotic and Systemic COPD, COPD = chronic obstructive pulmonary disease, COPDMAP = COPD Medical Research Council/Association of the British Pharmaceutical Industry, EndAECOPD = Endotype-Driven Prediction of Acute Exacerbations in COPD, HC = healthy control, IQR = interquartile range, IS = induced sputum, SS = spontaneous sputum

*2 weeks after therapy

†6 weeks post-exacerbation visit

‡The number of clean reads per sample included for downstream analysis for each dataset. Only one sample of less than 1000 clean reads was filtered during quality control for SRP066375 and BCCS & BCES, respectively

All datasets were sequenced at high depth (mainly on Miseq platform) with similar primers targeting V3-V5 hypervariable region of the 16S rRNA gene (Table 1) and processed separately using DADA2 algorithm [14] for data cleaning and taxonomic profiling. Despite this, hypervariable region and sequencing platform potentially lead to ASV-level heterogeneity [23]. Downstream microbiome analysis was therefore performed at genus level according to the MiBioGen consortium initiative [24] and previous experiences [23, 25], which sacrificed taxa classification resolution in exchange for less data heterogeneity.

### Microbial co-occurrence network in COPD stability reveals reduced negative interactions compared to HC

**Fig. 1:**
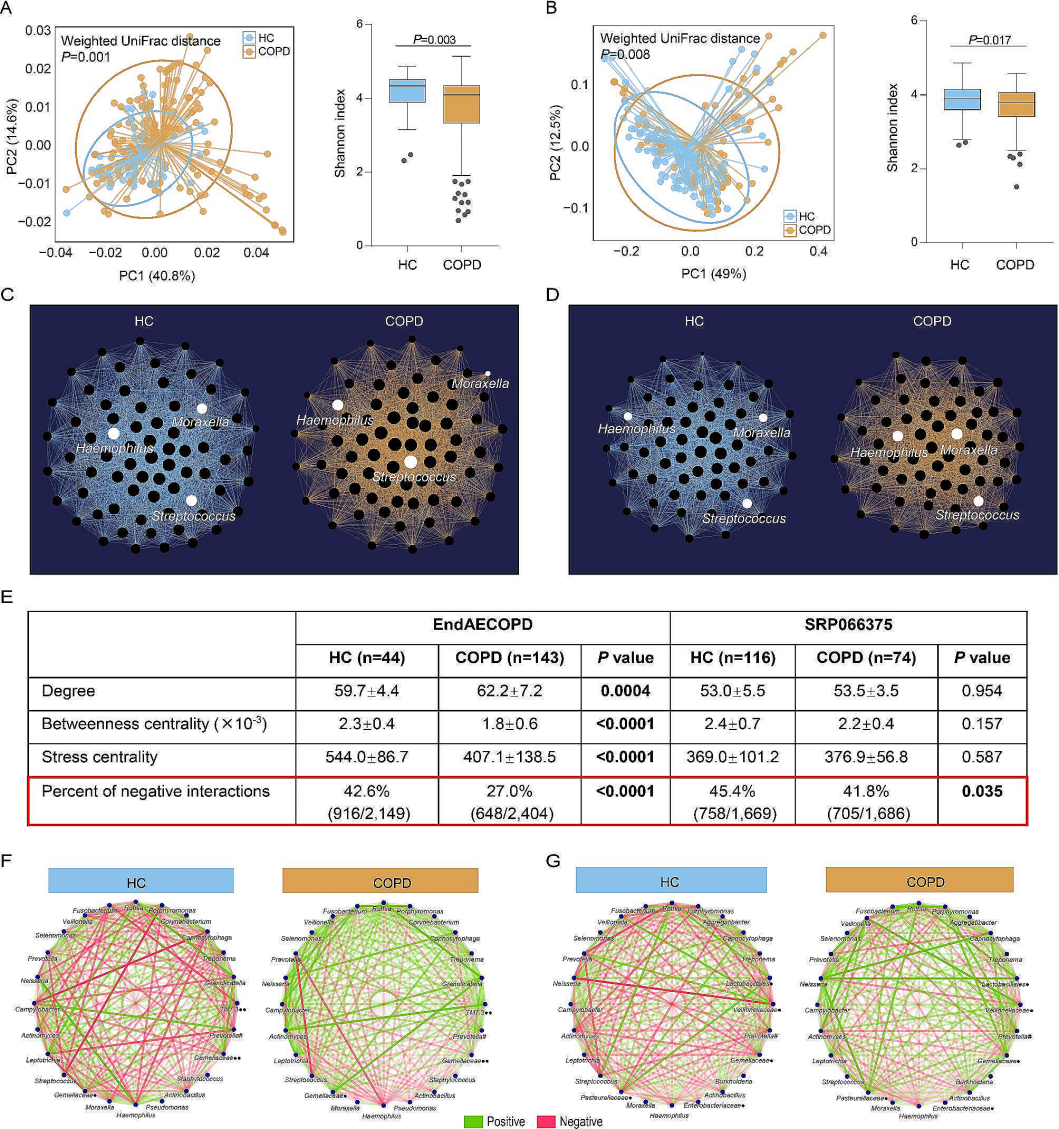
Co-occurrence network analysis of sputum microbiome between COPD and HC. **A, B**. Comparison of beta diversity (weighted UniFrac distance) and alpha diversity (Shannon index, Mann–Whitney *U*-test) between COPD and HC in the EndAECOPD cohort (*n* = 187) (**A**) and the SRP066375 cohort (*n* = 190) (**B**). **C, D**. Co-occurrence network maps of COPD and HC illustrating identified microbial interactions in EndAECOPD (**C**) and SRP066375 (**D**). Each node represents a microbe and microbial interactions are illustrated by connecting edges. The number of interactions for a microbe is calculated as degree. Node size is proportional to the number of interactions for each microbe. Selected taxa of clinical relevance are noted with white color. **E**. Comparison of key network parameters illustrating node and edge characteristics respectively between COPD and HC in both cohorts. Degree, betweenness centrality, and stress centrality were compared between COPD and HC by Mann–Whitney *U*-test. Percent of negative interactions, i.e. total number of negative interactions as a proportion of total interactions, were compared by *χ*^2^ test. **F, G**. Visualization of the opposing network by displaying positive and negative interactions between the most abundant taxa in COPD and HC in EndAECOPD (**F**) and SRP066375 cohort (**G**). Edge transparency is proportional to interaction strength between microbes in terms of edge weights. Negative interactions are classified if the sign of the edge weights is negative, and vice versa. # denotes paraphyletic group; • denotes unclassified genus; •• denotes classified but unnamed genus

We first performed health-COPD comparative analysis using data of the two Asian cohorts: EndAECOPD and SRP066375 (Table 1). Beta diversity analysis demonstrated a clear separation of samples between COPD and HC, and alpha diversity measured by Shannon index was reduced in COPD samples in both cohorts (Fig. 1A, B). We next applied weighted co-occurrence analysis with an ensemble of similarity measures [16, 17] to characterize potential interactions between different taxa (Fig. 1C, D). Network parameters reflecting node (microbe) importance including degree, betweennes centrality, and stress centrality showed substantial inconsistency across cohorts, which were significantly different between COPD and HC in EndAECOPD but not in SRP066375 (Fig. 1E). Yet when focusing on the sign of edge (microbial interaction), we found that patients with COPD in both cohorts had significantly reduced percent of negative interactions, i.e. total number of negative interactions as a proportion of total interactions, in their microbial network compared with HC (Fig. 1E-G). This trend remained significant when microbial networks were reconstructed separately by using either GBLM, Spearman, or Pearson correlation that determines the sign of edge weights in the ensemble network inference method in both cohorts (see Methods and Supplementary Table 6). To validate geographical reproducibility of this finding, we analyzed data of Haldar et al [26] that compared COPD (*N* = 218) versus HC (*N* = 124) sputum microbiomes separately retrieved from two UK cohorts. Again we observed significantly lower percent of negative interactions in microbial network between COPD versus HC (47.0%[616/1,311] versus 34.4%[188/547], *P* < 0.0001, *χ*^2^ test) even though COPD samples had significantly higher alpha diversity indices (Supplementary Fig. 2). Further appraisal of the effects of edge weights (interaction strength) on the observed change of negative interactions showed that the absolute weights of negative interactions were also decreased in COPD versus HC in all above data albeit not reaching statistical significance in SRP066375 (Supplementary Fig. 3). Collectively, we demonstrate that bacterial network disturbance characterized by reduced antagonistic interactions, rather than diversity loss, is a reproducible feature of COPD dysbiosis.

### Interactome analysis identifies a set of taxa whose antagonistic networks rather than abundances confer consistent changes in COPD

**Fig. 2:**
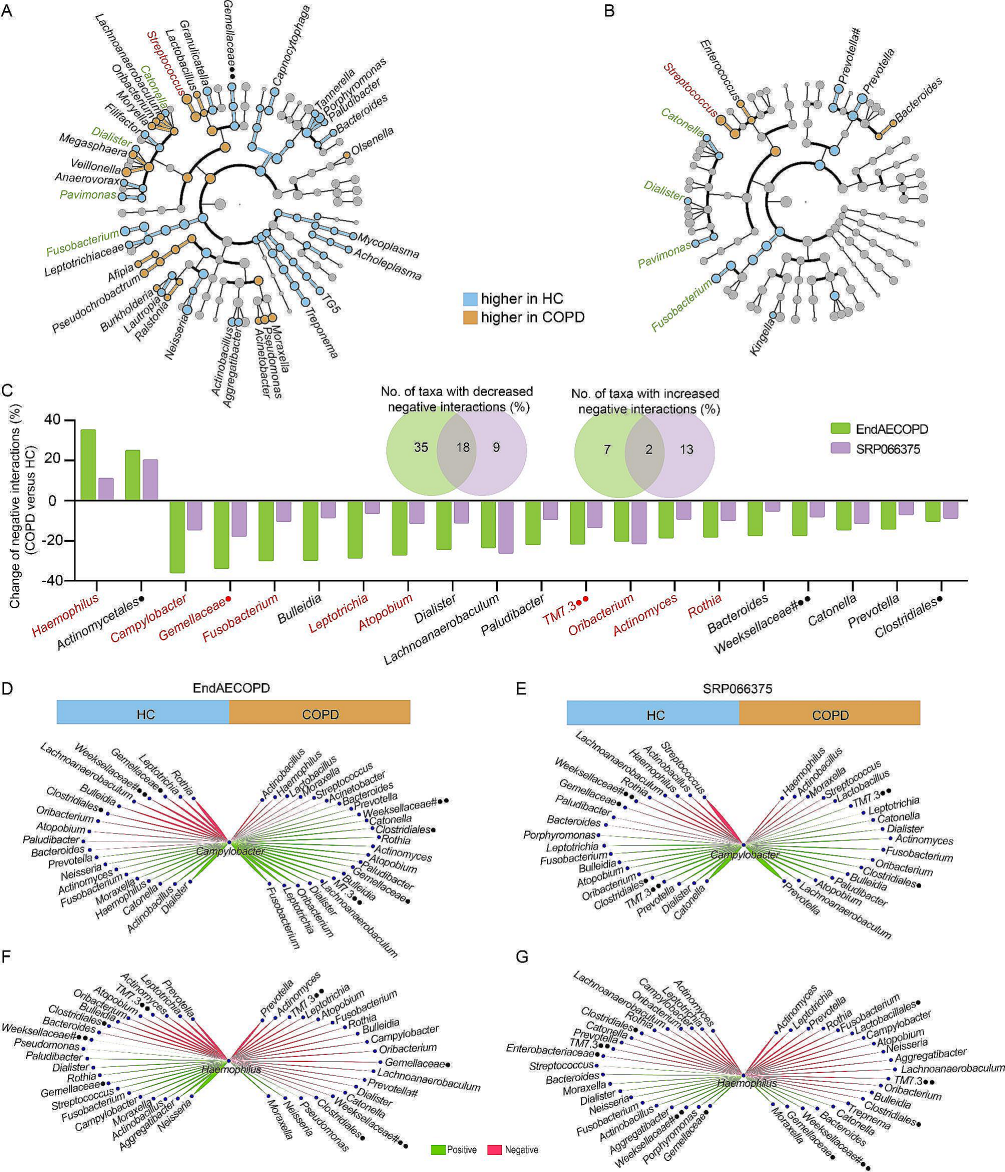
Network analysis of biomarker taxa in patients with COPD. **A, B** Enriched and depleted taxa in patients with COPD identified by Linear discriminant analysis effect size (LEfSe) analysis in EndAECOPD (**A**) and SRP066375 cohort (**B**). Genera significantly enriched or depleted in COPD in both cohorts were noted with red or green, respectively. (**C**) Venn diagram and barplot showing overlapped genera with altered degree of negative interactions (percent change of > 5%) between COPD versus HC in EndAECOPD and SRP066375 cohort. Genera that were further recovered in data of Haldar et al. were noted with red. (**D-G**). Visualization of the *Campylobacter* and *Haemophilus* interactomes between COPD and HC in EndAECOPD (**D, F**) and SRP066375 cohort (**E, G**). # denotes paraphyletic group; • denotes unclassified genus; •• denotes classified but unnamed genus

In general, differential abundance analysis is used to identify disease-associated microbial biomarkers, yet is sensitive to both technical artifacts and individual variabilities in environmental exposure and lifestyle [27]. This has led to the identification of varying biomarker species in different discovery cohorts, as suggested by EndAECOPD and SRP066375 datasets that have only a few overlapping taxonomic biomarkers (*Streptococcus, Catonella, Dialister, Pavimonas*, and *Fusobacterium*) (Fig. 2A, B). In contrast, through network analysis, we identified 20 taxa that showed consistently altered degree of negative interactions (percent change of > 5%) between COPD versus HC in the two cohorts (Fig. 2C), 10 of which (*Haemophilus*, *Campylobacter*, *Gemellaceae*, *Fusobacterium*, *Leptotrichia*, *Atopobium*, *TM7.3*, *Oribacterium*, *Actinomyces*, and *Rothia*) were also recovered in the aforementioned UK study by Haldar et al [26] (Supplementary Fig. 2). Most of these taxa (18/20) showed fewer negative interactions with other taxa in COPD, corresponding to the reduced antagonistic interactions of the entire network.

*Campylobacter*, recently found to be a strongest microbial biomarker of eosinophilic COPD, was the top contributor to the decreased network antagonism in COPD, while *Haemophilus*, an established neutrophilia-associated pathogen, acted in the opposite way [3] (Fig. 2C). Changes in relative abundance of these two genera in COPD versus HC were neither observed in most of previous microbiome studies (Supplementary Table 1) nor in EndAECOPD and SRP066375 cohorts (Fig. 2A, B; Supplementary Fig. 4). In comparison, our network analysis identified distinct *Campylobacter* and *Haemophilus* interactomes regarding proportion of negative interactions between COPD and HC in both cohorts (Fig. 2D-G), suggesting the superiority of interactomics over differential abundance analysis in the identification of disease-associated biomarkers.

### Airway microbial network displays reduced negative interactions during COPD exacerbation, which recovers following treatment

**Fig. 3:**
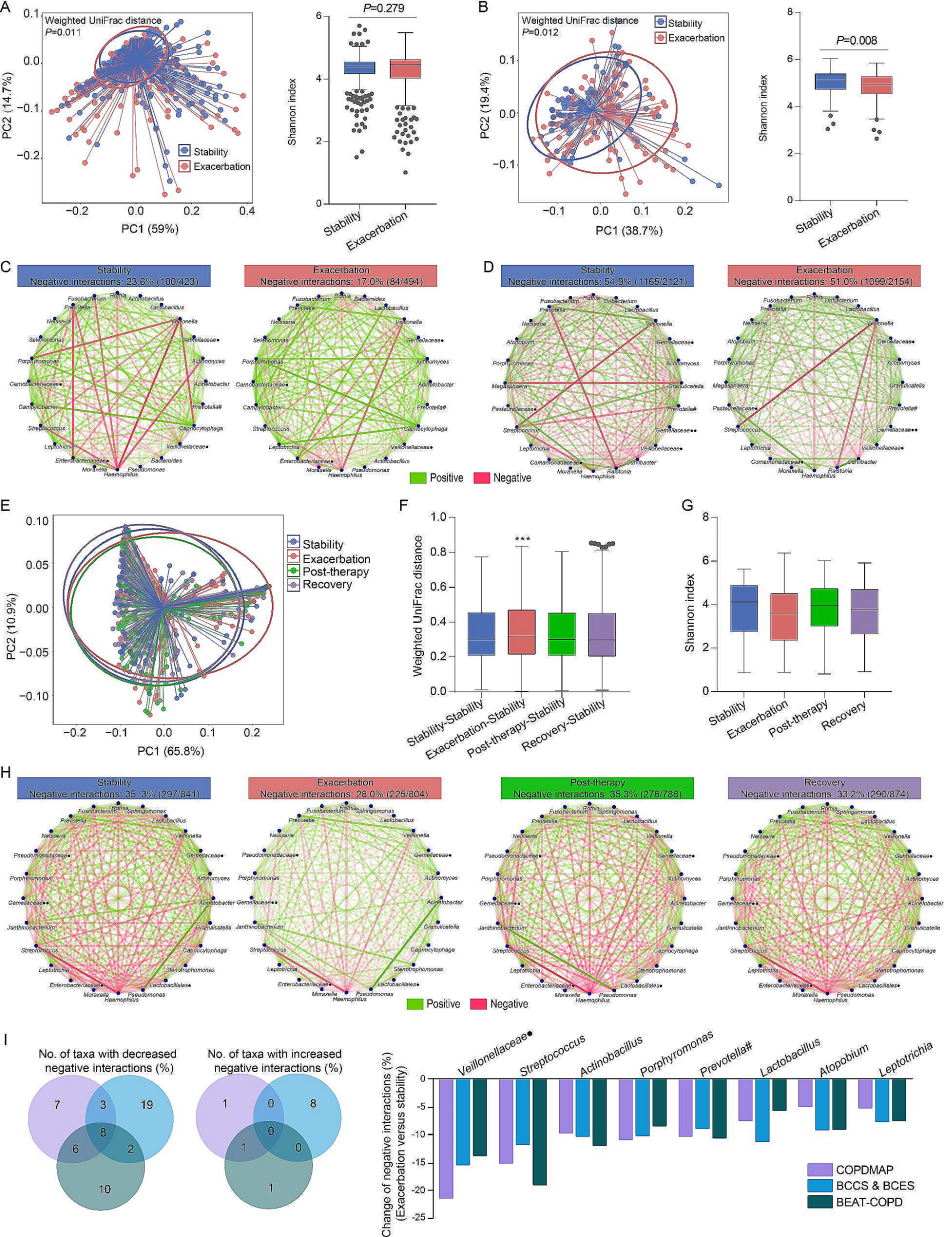
Longitudinal analysis of the COPD interactome during exacerbations. (**A, B**). Comparison of beta diversity (weighted UniFrac distance) and alpha diversity (Shannon index, Mann–Whitney *U*-test) between stability and exacerbation samples in COPDMAP (*n* = 715) (**A**) and BCCS & BCES cohort (*n* = 174) (**B**). (**C, D**). Visualization of the interactomes in terms of positive and negative interactions between the most abundant taxa at stability and during exacerbation in COPDMAP (**C**) and BCCS & BCES (**D**). # denotes paraphyletic group; • denotes unclassified genus; •• denotes classified but unnamed genus. (**E, F**). Microbial clustering (**E**) and one-way ANOVA analysis of weighted UniFrac distance for beta diversity (**F**) between stability, exacerbation, post-therapy, and recovery in the BEAT-COPD cohort (*n* = 476). *** *P* < 0.0001 (Exacerbation-stability distance versus Stability-stability distance). (**G**). One-way ANOVA analysis of alpha diversity (Shannon index) between stability, exacerbation, post therapy, and recovery in BEAT-COPD cohort. (**H**). Visualization of the interactomes in terms of positive and negative interactions between the most abundant taxa across timepoints in BEAT-COPD cohort. (**I**). Venn diagram and barplot showing overlapped genera with altered degree of negative interactions (percent change of > 5%) between exacerbation versus stability across the three cohorts

To clarify how bacterial interactomes would change during the course of COPD exacerbation, we performed stability-exacerbation comparative analysis using data of COPDMAP and BCCS & BCES cohorts (Table 1). Beta diversity analysis clearly distinguished samples between stability and exacerbation in the two cohorts, however, the change in Shannon index was incongruent (Fig. 3A, B). In contrast, we observed significantly lower degree of antagonistic interactions in microbial network during exacerbations of COPD in both COPDMAP (*P* = 0.012) and BCCS & BCES (*P* = 0.011) (Fig. 3C, D). We further evaluated the dynamic change of COPD interactomes at four clinical timepoints: stability, exacerbation, post-therapy (2 weeks after treatment), and recovery (6 weeks post-exacerbation) in the BEAT-COPD cohort. Longitudinal analysis revealed the deviation of exacerbation microbiomes in beta-diversity to other timepoints (Fig. 3E, F), demonstrating the presence of dysbiosis at exacerbation. However, such dysbiosis was not captured by alpha diversity analysis (Fig. 3G). By contrast, co-occurrence network analysis highlighted major changes in interactomes, with a decrease in percent of negative microbial interactions during exacerbations compared with baseline (*P* = 0.001), post-therapy (*P* = 0.002), or at recovery (*P* = 0.021) (Fig. 3H). Further breakdown of interactomes identified eight taxa (*Veillonellaceae*, *Streptococcus*, *Actinobacillus*, *Porphyromonas*, *Prevotella*[paraphyletic group], *Lactobacillus*, *Atopobium*, and *Leptotrichia*) with decreased negative interactions during exacerbation across COPDMAP, BCCS & BCES, and BEAT-COPD (Fig. 3I). These findings highlight microbial interactome disturbance rather than diversity loss as a reproducible feature of COPD exacerbation.

### Airway microbial interactome is associated with key clinical determinants of COPD

**Fig. 4:**
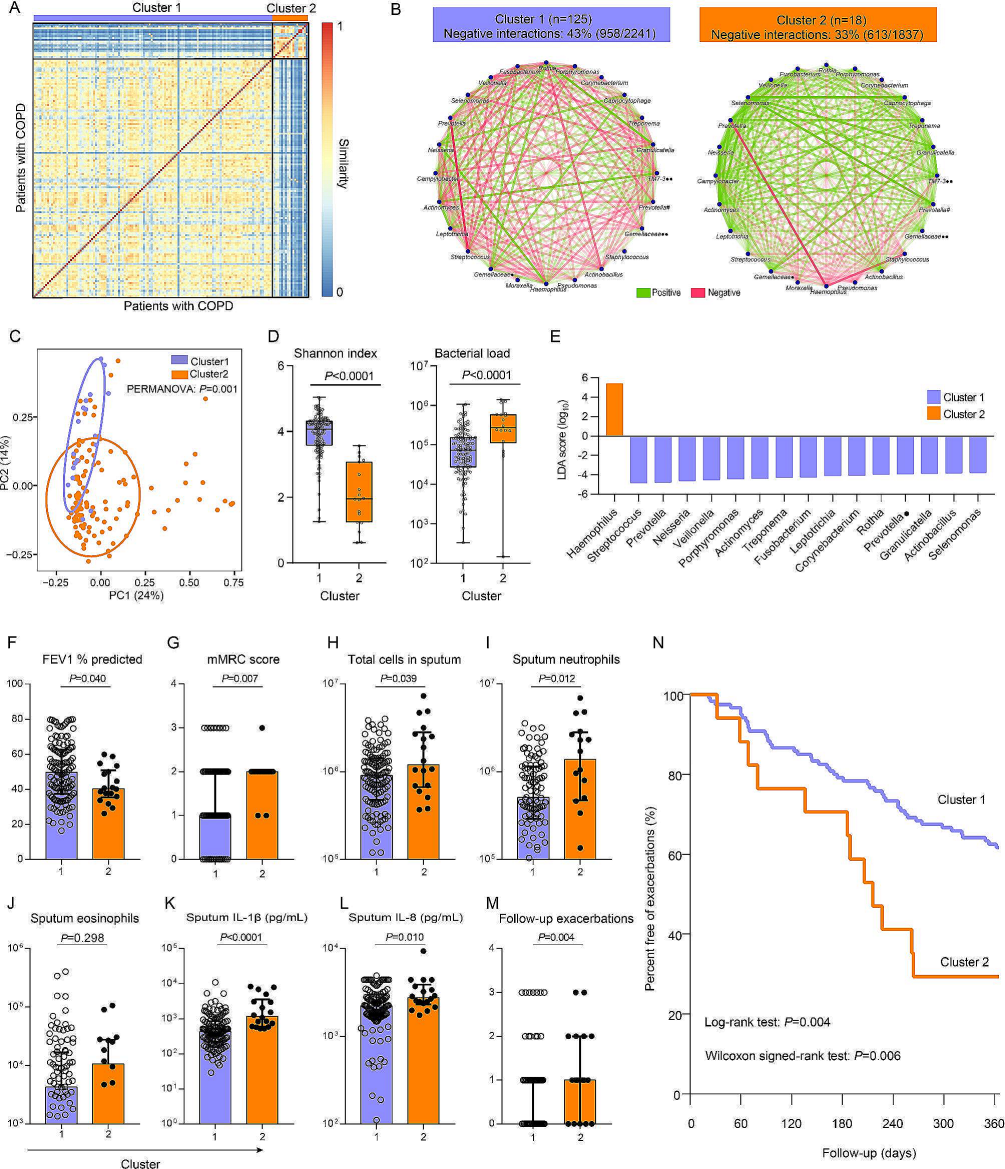
Clinical determinants correlate with microbial network. (**A**) Unsupervised stratification of microbial networks with spectral clustering for the 143 COPD patients in EndAECOPD cohort into two clusters (cluster 1, *n* = 125; cluster 2, *n* = 18). (**B**). Visualization of positive and negative interactions between the most abundant taxa in each cluster. Cluster 2 had significantly lower proportion of negative bacterial interactions compared with cluster 1 (*P* < 0.0001, *χ*^2^ test). # denotes paraphyletic group; • denotes unclassified genus; •• denotes classified but unnamed genus. (**C**). Principal Coordinates Analysis (PCoA) illustrating beta diversity (Weighted UniFrac distance) between network clusters. (**D**). Comparison of Shannon index and bacterial load (16S rRNA gene copies/uL sputum plug) betweeen clusters (Mann–Whitney *U*-test). (**E**). LEfSe analysis of differentially abundant taxa between clusters. (**F-L**). Comparison of baseline lung function (FEV1% predicted) (**F**), breathlessness (mMRC) score (**G**), and sputum total cells (**H**), neutrophils (**I**), eosinophils (**J**), IL-1β (**K**), and IL-8 (**L**) between the two identified patient clusters. (**M**). Comparison of follow-up moderate-to-severe exacerbations between the two clusters. (**N**). Survival analysis of differences between the two clusters in the time to first exacerbation

Through unsupervised spectral clustering on the baseline sputum microbial network of COPD patients in EndAECOPD cohort, we identified two network clusters (Fig. 4A), with a cluster robustness of 99%. The cluster 2 (*n* = 18) had significantly lower proportion of negative bacterial interactions compared with the cluster 1 (*n* = 125) (Fig. 4B). Differences in microbial compositions were also evident between the two clusters as shown in Fig. 4C-E. Demographics including age, sex, smoking history, and medication use were similar between the two clusters except that patients in cluster 2 had lower body mass index (*P* = 0.035) (Supplementary Table 7). In assessment of COPD-related parameters, patients in cluster 2 were found to have lower lung function (FEV1% predicted) (*P* = 0.040) and higher mMRC score (*P* = 0.007) (Fig. 4F, G). Further evaluation of airway inflammatory status by profiling sputum cells and a panel of 19 cytokines revealed that patients in cluster 2 had exaggerated neutrophilic inflammation in terms of sputum total cells, neutrophil counts, interleukin (IL)-1β and IL-8 levels (Fig. 4H-L). During the 12-month follow-up, 71%[12/17] of patients in cluster 2 experienced an exacerbation while only 38%[46/120] of patients in cluster 1 had an exacerbation (*P* = 0.012). Patients in cluster 2 experienced significantly more moderate-to-severe exacerbations (*P* = 0.004) (Fig. 4M) and had higher proportion of frequent exacerbators (35%[6/17] versus 11%[13/120], *P* = 0.015). A life-table analysis showed that the exacerbation risk was significantly higher among patients in cluster 2 (Log-rank test, *P* = 0.004; Wilcoxon signed rank test, *P* = 0.006) (Fig. 4N).

### Frequent exacerbators have reduced antagonistic interactions in microbiota while exhibiting subtle compositional changes

**Fig. 5:**
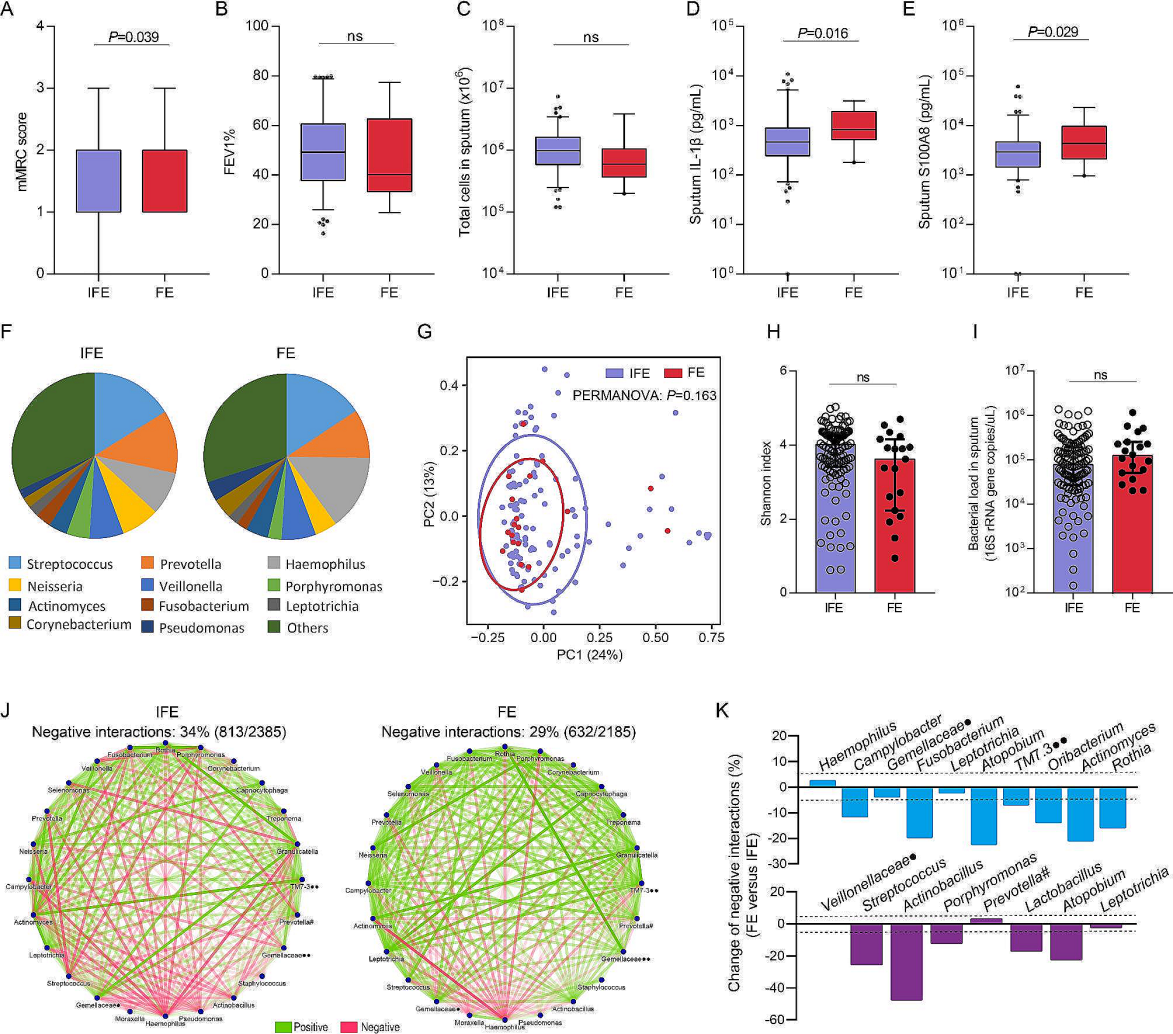
Network analysis of the interactome in frequent exacerbators. Classification of the COPD patients in the EndAECOPD cohort according to exacerbator phenotype. (**A-E**). Comparison of breathlessness (mMRC) score (**A**), lung function (FEV1% predicted) (**B**), sputum total cells (**C**) and cytokines (**D, E**) between exacerbator phenotypes. Of the 19 detected cytokines, IL-1β (**D**) and S100A8 (**E**) were significantly elevated in frequent exacerbators. Box plots reflect median and IQRs, with whiskers (5–95 percentile) bounding non-outlier values. ns, not significant (Mann–Whitney *U*-test). IFE = infrequent exacerbator, FE = frequent exacerbator. **F**. Barplots of microbial abundance displaying similar taxonomic composition between exacerbator phenotypes which was confirmed by LEfSe analysis revealing no differentially abundant taxa. (**G**). PCoA illustrating similar beta diversity (Weighted UniFrac distance) between exacerbator phenotypes. (**H, I**). Comparison of Shannon index (**H**) and bacterial load (**I**) between exacerbator phenotypes with Mann–Whitney *U*-test. (**J**). Opposing network analysis of exacerbator phenotypes. The frequent exacerbators had significantly lower proportion of negative interactions in microbial network compared with infrequent exacerbators (*P* < 0.0001, *χ*^2^ test). # denotes paraphyletic group; • denotes unclassified genus; •• denotes classified but unnamed genus. K. Change of negative interactions (%) for the stability-related taxa (blue, up) and exacerbation-related taxa (purple, down) identified in above network analysis in frequent versus infrequent exacerbators. The dashed line represents ± 5%

Finally, we focused specifically on the association of microbial network with the frequent exacerbator phenotype (≥ 2 moderate-to-severe exacerbations during 1-year follow-up). We classified COPD patients according to exacerbator phenotype (*n* = 137; 6 patients lost to follow-up) in EndAECOPD. Characteristics between frequent and infrequent exacerbators were shown in Supplementary Table 8. Frequent exacerbators (*n* = 19) had significantly greater breathlessness (mMRC) score and higher level of sputum IL-1β (*P* = 0.016) and S100A8 (*P* = 0.029) compared to infrequent exacerbators (*n* = 118) (Fig. 5A–E). Conventional microbiome analysis indicated a broad similarity of microbiome signatures, with no significant differences observed in microbial composition, beta diversity, alpha diversity or bacterial load (Fig. 5F–I). Yet interestingly, the frequent exacerbator phenotype presented the typical network disturbance, i.e., reduced antagonistic interactions (Fig. 5J). Besides, 7 of the 10 stability-related taxa and 5 of the 8 exacerbation-related taxa identified in above network analysis also showed reduced negative interactions in frequent exacerators (Fig. 5K). These findings suggest that network disturbance, instead of alterations in microbial abundance or load, may be a key driver of the frequent exacerbator phenotype in patients with COPD.

## Disscussion

In this paper, we have performed a cross-cohort microbiome analysis to uncover the interaction pattern of airway bacteria in COPD. We demonstrate that loss of negative bacterial interactions, rather than change in microbial abundance or diversity, is a reproducible feature of COPD dysbiosis both at clinical stability and during exacerbation, which is strongly associated with disease phenotypes especially exacerbation risk.

This study reveals a highly reproducible signature of COPD network disturbance, that is, reduced negative interactions, which is validated in geographically diverse cohorts across different disease phases regardless of using different network inference methods. Interpretation of its ecological relevance requires further experimental discoveries, as computationally interspecies relationships can have various ecological meanings [28]. Among these, cooperation (positive) and competition (negative) are two commonest types of microbial interactions [29]. Growing evidence demonstrate that cooperating networks are ecologically unstable due to positive feedbacks whereas microbial competitions suppress these positive feedbacks to promote microbiota stability that is essential for host health [29, 30]. Therefore, the observed decrease in proportion of negative bacterial interactions in COPD may reflect an imbalance between cooperation and competition within ecological networks where reduced competition leads to dysregulated positive feedbacks and subsequent microbiota destabilization. Such unstable microbiome communities are marked by their vulnerability to perturbations [29], which potentially underpins the clinical observations that patients with COPD easily develop airway dysbiosis and deteriorated respiratory symptoms (exacerbation) upon exposure to various irritants [31]. Indeed, we detected significant perturbations of microbiota during COPD exacerbation in three sizable cohorts, as indicated by fewer antagonistic interactions and distinct beta diversity. Intriguingly, although unstable, the microbial interactome in COPD is somewhat resilient and plastic, as suggested by the longitudinal interactome dynamics in which negative interactions decreased during exacerbation but recovered after treatment. Collectively, loss of negative microbial interactions is likely an indicator of competitive deficiency and microbiota destabilization in COPD that is potentially modifiable.

The interactomics provides a new conceptual framework for understanding the microbial etiology of COPD and its exacerbation. Traditionally, chronic infection or colonization with pathogenic bacteria especially *Haemophilus* is thought to promote chronic airway inflammation and an increase in bacterial load triggers exacerbations [32]. However, this theory does not consider coexisting commensals that may interact with respiratory pathogens. Here interactomic analyses suggest that the persistent airway inflammation of COPD that exaggerates during exacerbation is more likely to be a consequence of bacterial interactome disturbance rather than infection. This is first supported by our analysis of the *Haemophilus* interactome showing that the interaction pattern of this established pathogen rather than its abundance is altered in COPD. Besides, the interactome disturbance featured by reduced negative interactions is prevalent both at COPD stability and during exacerbation while diversity loss, a sign of bacterial infections, is relatively rare [33]. Additionally, we verify in the EndAECOPD cohort that the interactome disturbance is correlated with COPD neutrophilic inflammation and exacerbation events (Fig. 4). Although *Haemophilus* was also enriched in the patient cluster characterized by interactome disturbance (Fig. 4E), indicating a potential link between *Haemophilus* colonization with airway inflammation and exacerbation. We showed in the exacerbator phenotype analysis that frequent exacerbators, characterized by greater mMRC score and higher level of sputum IL-1β, had reduced antagonistic interactions in microbiota while exhibiting no significant change in microbial abundance, diversity, and bacterial load (Fig. 5). These data suggest that network disturbance, rather than alterations in microbial abundance or load, may be a key driver of airway inflammation and exacerbations in patients with COPD.

COPD is a well-recognized phenotypically-heterogeneous disease, which is being addressed by delineating the shared biological underpinnings [34]. In this work, we have identified clinical correlations between bacterial interactome and multiple key disease parameters including lung function, symptom score, airway inflammation, and exacerbation risk. Furthermore, when focusing specifically on the frequent exacerbator phenotype, that is, one of the most important COPD phenotypes, we showed that the frequent exacerbators had distinct microbial interactomes but no difference in microbiota compositions, suggestive of microbial network rather than abundance alone that potentially drives this clinical state. In line with this, a previous large-scale microbiome study also showed no clear clustering of microbiome between exacerbator phenotypes according to beta diversity metrics [4]. Together with the finding that interactomes differ between COPD and HC and show dynamic change during exacerbations, this study highlights a clinical importance of microbial interactome in COPD especially the exacerbator phenotype which may help identify risk, guide therapy, or be targeted itself for outcome improvement.

The findings of this work provide new insights for design of strategies to modify airway dysbiosis. Long-term antibiotic treatment, such as macrolides, has been shown to modify the airway microbiome, however, it does not effectively eradicate common airway pathogens either in emphysema, COPD, or bronchiectasis [35–37]. The identification of a disturbed microbial interactome in COPD that is potentially plastic suggest that we may target interactome rather than pathogen alone for disease treatment. Increasing evidence demonstrate that enhancing commensal competitiveness by supplementation of probiotics can promote intestinal pathogen elimination [38] and increase microbiota stability [39]. Here our interactome analysis identifies 18 genera (*Campylobacter*, *Gemellaceae*•, *Fusobacterium*, *Bulleidia*, *Leptotrichia*, *Atopobium*, *Dialister*, *Lachnoanaerobaculum*, *Paludibacter*, *TM7.3*••, *Oribacterium*, *Actinomyces*, *Rothia*, *Bacteroides*, *Weeksellaceae*#••, *Catonella*, *Prevotella*, and *Clostridiales*•) that show consistently reduced negative interactions in COPD, suggesting their potential role in determining microbiota stability. To note, most of the 18 genera characterized by reduced negative interactions, e.g., *Campylobacter*, *Fusobacterium*, *Leptotrichia*, *Prevotella*, *Catonella*, *Dialister*, *Lachnoanaerobaculum*, *Oribacterium*, *Actinomyces*, *Rothia*, *Bacteroides*, have been reported to be depleted in COPD in the current and previous studies [23, 26, 40]. Besides, genera like *Rothia* have been experimentally confirmed to exert anti-inflammatory effects in a *Pseudomonas aeruginosa* infection model [10]. Therefore, the identified 18 genera may be potentially beneficial. Furthermore, these commensals generally show negative interactions with *Haemophilus* in COPD (Fig. 2F, G), which may imply their broad competition with this pathogen. Future studies are needed to test whether these commensals may potentially be used as probiotics to eliminate respiratory pathogens and rebuild microbiota stability in COPD.

Our study has several limitations. We included both induced and spontaneous sputum samples for assessment of exacerbation interactomes. This is a potential confounder although previous analyses suggested no significant differences in the microbiota between the two types of samples [41]. It is also uncertain how sputum can recapitulate the airway ecology given its inherent mixture of materials from the upper and lower respiratory tracts. The absence of adequate negative controls in publicly available datasets may limit the robustness of the analyses conducted. Aggregating data to the genus level leads to a loss of resolution, potentially obscuring important taxonomic distinctions. Except for bacteria, viruses and fungi are key members of airway microbiota and inter-kingdom interactions may also play crucial role in chronic airway diseases [17] which deserve further investigation. The associations of bacterial network with COPD phenotypes were only evaluated in a single cohort with moderate sample size, thus require external validations. While network-based inference methods offer valuable insights into microbial interactions, their direct translation to clinical practice may be challenging. Especially in the context of the respiratory tract where bacterial abundance is typically low, the relevance and applicability of inferred networks to real-life interactions between bacteria may be limited. Besides, the observed bacterial interactions may potentially be affected by host responses but have not been adequately assessed in our work. Future large-scale longitudinal metagenomic study with host multiomics profiling is needed to bring in a more comprehensive picture of microbial interacting network and its implications in disease.

The cross-cohort microbiome analysis identifies loss of negative bacterial interactions as a reproducible feature of COPD airway dysbiosis both at clinical stability and during exacerbation, and as a strong predictor of exacerbation risk. These findings expand our current understanding of COPD dysbiosis based on differentially abundant taxa or diversity metrics, and provide insight for interventional studies to restore airway ecosystems by means other than antibiotics, e.g. probiotics supplementation.

### Electronic supplementary material

Below is the link to the electronic supplementary material.

Supplementary Material 1

## Data Availability

The dataset and metadata generated during the current study is available in the Genome Sequence Archive in National Genomics Data Center (HRA003966) at https://ngdc.cncb.ac.cn/gsa-human/browse/HRA003966. Public microbiome datasets used in this manuscript are downloaded from the National Center for Biotechnology Information Sequence Read Archive (SRP066375, SRP102480, SRP065072), the National Centre for Biotechnology Information (PRJNA491861), and the Dryad Digital Repository (Dryad.5gc82). Analytical codes and scripts used for analysis are available at https://github.com/weifly000/COPD-interactome.
