# Supplementary material for: Bacterial interactome disturbance in chronic obstructive pulmonary disease clinical stability and exacerbations
Source: Respir Res. 2024 Apr 20;25:173. doi: 10.1186/s12931-024-02802-5 (PMC11032604; doi:10.1186/s12931-024-02802-5)
Supplement: Supplementary file 1 — Supplementary Material 1 [file 12931_2024_2802_MOESM1_ESM.docx]

**Bacterial interactome disturbance in chronic obstructive pulmonary disease clinical stability and exacerbations**

Wei Xiao, Yi-long Chen, Long-yi Du, Jiqiu Wu, Zhang Wang, Bing Mao, Fu-qiang Wen, Peter Gerard Gibson, Vanessa M. McDonald, Haopeng Yu, Juan-juan Fu

**ON LINE DATA SUPPLEMENT**

**SUPPLEMENTARY METHODS**

Reagent controls

For EndAECOPD cohort, reagent controls for extraction (no sputum material) and PCR amplification (no DNA template) were included. Gel electrophoresis of PCR products showed that sputum samples had clear bands against marker bands while reagent control samples did not (Supplementary Figure 1), suggesting that reagent contamination was not introduced during this process.

**SUPPLEMENTARY TABLES**

**Supplementary Table 1. Summary of case-control studies comparing airway microbiome between COPD and healthy control (HC)**

| **Author** | **Year** | **COPD** | **HC** | **Sample type** | **Country** | **Platform** | **Primer** | **Conclusions** |
| --- | --- | --- | --- | --- | --- | --- | --- | --- |
| Hilty[1] | 2010 | 5 | 8 | BB | Ireland | 454 | V3-5 | Differing β diversity |
| Erb[2] | 2011 | 4 | 10 | BAL | USA | 454 | V1-3 | Lack of power to draw a conclusion |
| Pragman[3] | 2012 | 22 | 10 | BAL | USA | 454 | V3 | Increased α diversity, differing β diversity, and increased *Fusobacteria*, Campylobacter, and *Lactobacillales* |
| Sze[4] | 2012 | 8 | 16 | LT | Canada | 454 | V2 | Similar α diversity, differing β diversity, and increased *Firmicutes* (*Lactobacillus*) |
| Molyneaux[5] | 2013 | 14 | 17 | IS | UK | 454 | V3-5 | Similar α diversity, similar β diversity, decreased *Proteobacteria*, and increased  *Firmicutes (Veillonellaceae)* |
| Sze[6] | 2015 | 5 | 4 | LT | Canada | 454 | V3-5 | Decreased α diversity, differing β diversity, increased *Proteobacteria*, but decreased  *Haemophilus* |
| Yu[7] | 2016 | 74 | 91 | LT* | Italy | Miseq | V3-4 | Similar α diversity, β diversity, and *Proteobacteria* |
| Einarsson[8] | 2016 | 18 | 19 | BAL | UK | MiSeq | V1-2 | Decreased α diversity, differing β diversity, decreased *Bacteroidetes* (*Prevotella*), and similar *Proteobacteria* (*Haemophilus*) |
| Kim[9] | 2017 | 13 | 26 | LT | Korea | 454 | V1-3 | Decreased α diversity, differing β diversity, and increased *Firmicutes* (*Lactobacillales*) |
| Engel[10] | 2017 | 16 | 9 | BB | Europe† | 454 | V6-9 | Similar α diversity, similar β diversity, increased *Streptococcus*, and decreased *Prevotella* |
| Diao[11] | 2018 | 40 | 19 | IS | China | 454 | V3-5 | Similar β diversity |
| Wang[12] | 2019 | 43 | 16 | SS&IS | UK | Miseq | V4 | Decreased α diversity, and increased *Moraxella*, *Streptococcus* and *Actinobacteria* |
| Haldar[13]‡ | 2020 | 218 | 124 | SS&IS | UK | Miseq | V4 | Increased α diversity, differing β diversity, increased *Gammaproteobacteria*, and *decreased Firmicutes, Bacteroidetes* and *Actinobacteria* |
| Ramsheh[14] | 2021 | 339 | 207 | BB | Europe§ | Miseq | V4-5 | Decreased α diversity, similar β diversity, decreased *Prevotella*, increased *Streptococcus*, similar *Haemophilus* and *Veillonella* |

BB=Bronchial brush, EA=Endotracheal aspirate, LT=lung tissue, IS=induced sputum, SS=spontaneous sputum. *Non-malignant lung tissue; †including nine centers in the UK, Germany, Italy, Poland, and Hungary; ‡This study compared COPD versus HC sputum microbiomes from separate cohorts; §including Germany, Italy, UK, and Hungary.

**Supplementary Table 2. Summary of studies comparing airway microbiome between COPD clinical stability and exacerbation**

| **Author** | **Year** | **stable** | **ex** | **Sample type** | **Country** | **Platform** | **Primer** | **Conclusions** |
| --- | --- | --- | --- | --- | --- | --- | --- | --- |
| Millares[15] | 2015 | 8 | 8 | SS | Spain | 454 | V1-V3 | Similar α diversity, similar β diversity |
| Wang[16] | 2016 | 106 | 137 | SS | UK | 454 | V3-V5 | Reduced α diversity, and increased Moraxella |
| Garcia-Nuñez[17] | 2017 | 14 | 14 | SS | Spain | 454 | V1-3 | Differing β diversity |
| Wang[18] | 2018 | 446 | 270 | SS | UK | Miseq | V4 | Similar α diversity, similar β diversity, and decreased *Veillonella* |
| Mayhew[19] | 2018 | 423 | 161 | SS&IS | UK | Miseq | V4 | Similar α diversity, increased Moraxella |
| Jubinville[20] | 2018 | 9 | 9 | SS&IS | Canada | 454 | V6-V8 | Lack of power to draw a conclusion |
| Tangedal[21] | 2019 | 36 | 36 | IS | Bergen,  Norway | MiSeq | V3-4 | Similar α diversity, differing β diversity |
| O’Farrell[22] | 2019 | 14 | 24 | SS | Australia | Miseq | V1-V3 | Similar α diversity, similar β diversity, increased Pseudomonas |
| Wang[12] | 2019 | 32 | 16 | SS&IS | UK | Miseq | V4 | Decreased α diversity, increased *Moraxella* |
| Bouquet[23] | 2020 | 447 | 396 | SS | Europe,  USA | Miseq | V4 | Similar α diversity |
| Wang[24] | 2020 | 4 | 36 | SS&IS | China | Hiseq | V3-V4 | Decreased α diversity(not statistically), differing β diversity |
| Goolam[25] | 2021 | 18 | 6 | SS | South Africa | Miseq | V1-V3 | Similar α diversity, similar β diversity |
| Su[26] | 2022 | 23 | 28 | SS&IS | China | Novaseq | V3-V4 | Decreased α diversity, differing β diversity，increased Proteobacteria and Actinobacteria, and decreased Firmicutes and Bacteroidetes. |

IS=induced sputum; SS=spontaneous sputum.

**Supplementary Table 3. The search term and the inclusion/exclusion criteria.**

| **Search term** | Pubmed | (16S OR microbiota OR microbiome) AND (chronic obstructive pulmonary disease OR COPD) AND sputum |
| --- | --- | --- |
|  | SRA database | (chronic obstructive pulmonary disease OR COPD) AND sputum AND "Homo sapiens"[orgn:__txid9606] |
|  | ENA database | (chronic obstructive pulmonary disease OR COPD) AND sputum |
| **Inclusion/exclusion criteria** | Inclusion criteria | (1) Case-control studies of patients with stable COPD *versus* healthy controls or cohort studies of COPD stability *versus* exacerbation;  (2) Available sputum samples;  (3) Available 16S rRNA gene sequencing data with grouping information in metadata file. |
|  | Exclusion criteria | (1) Subjects had other respiratory diseases;  (2) Stable COPD patients or healthy controls received antibiotic use within the past month;  (3) Sample size less than 20 for an individual group after data processing and quality control. |

SRA=the Short Read Archive; ENA=the European Nucleotide Archive.

**Supplementary Table 4. Demographics of the EndAECOPD cohort.**

|  | **Total** | **HC** | **COPD** | ***P* value** |
| --- | --- | --- | --- | --- |
| No. of subjects | 187 | 44 | 143 |  |
| Age (IQR) | 63(51–68) | 47(45–51) | 65(61–70) | <0.0001 |
| Male | 130(70%) | 13(30%) | 117(82%) | <0.0001 |
| BMI (kg/m^2^) | 23.4±2.9 | 24.5±2.1 | 23.0±3.0 | 0.003 |
| Current smoker | 25(13%) | 4(9%) | 21(15%) | 0.340 |
| Ex-smoker | 102(55%) | 6(14%) | 96(67%) | <0.0001 |
| Pack years | 24(0–42) | 0(0–0) | 32(14–48) | <0.0001 |
| Pulmonary function |  |  |  |  |
| FEV_1_ (L) | 1.61(1.10–2.10) | 2.33(2.07–2.75) | 1.33(1.04–1.75) | <0.0001 |
| FVC (L) | 2.84(2.35–3.36) | 2.88(2.50–3.30) | 2.79(2.23–3.37) | 0.282 |
| FEV_1_/FVC (%) | 53.4(44.4–69.8) | 82.0(78.3–86.0) | 49.8(42.8–57.0) | <0.0001 |
| FEV_1_ % predicted | 55.3%(40.6–76.5) | 87.1(79.3–94.0) | 48.7(36.8–61.8) | <0.0001 |
| CAT score |  |  | 11(7–15) |  |
| mMRC score |  |  | 2(1–2) |  |
| Exacerbations in prior year (≥2) |  |  | 41(29%) |  |
| Hospitalized exacerbations in prior year (≥1) |  |  | 56(39%) |  |
| GOLD 2020 group |  |  |  |  |
| A |  |  | 23 (16%) |  |
| B |  |  | 54 (38%) |  |
| C |  |  | 14 (10%) |  |
| D |  |  | 53 (37%) |  |
| Charlson comorbidity index |  |  | 3(3–4) |  |
| Medication use |  |  |  |  |
| ICS+LABA+LAMA |  |  | 69 (48%) |  |
| ICS+LABA |  |  | 31 (22%) |  |
| LAMA only |  |  | 18 (13%) |  |
| Sputum total cell counts (×10^6^/mL) |  |  | 0.93(0.51–1.46) |  |
| Sputum differential cell counts (%) |  |  |  |  |
| Neutrophils |  |  | 71.8(49.6–87.8) |  |
| Macrophages |  |  | 22.0(9.6–42.2) |  |
| Lymphocytes |  |  | 1.3(0.5–2.6) |  |
| Eosinophils |  |  | 0.5(0.0–1.8) |  |
| Epithelial cells |  |  | 0.4(0.0–1.4) |  |

BMI=Body mass index; CAT=COPD Assessment Test; FEV_1_=Forced expiratory volume in the first second; FVC=Forced vital capacity; IQR=interquartile range; ICS=inhaled corticosteroids; LABA=Long-acting beta-agonist; LAMA=Long‐acting muscarinic antagonist; mMRC= Modified Medical Research Council Dyspnea Scale.

**Supplementary Table 5. The quality control statistics of the 16S datasets.**

| **Datasets** | **Total reads** | **Reads passing quality controls** | **Non-chimera reads** | **Ratio of reads passing quality control** | **Ratio of non-chimera reads** |
| --- | --- | --- | --- | --- | --- |
| EndAECOPD | 9783434 | 9624383 | 9042593 | 0.984 | 0.924 |
| SRP066375 | 7408247 | 7240743 | 6752490 | 0.977 | 0.911 |
| COPDMAP | 46805924 | 45788293 | 44782149 | 0.978 | 0.957 |
| BCCS & BCES | 11437843 | 11327696 | 10446147 | 0.990 | 0.913 |
| BEAT-COPD | 5303124 | 5126191 | 4646662 | 0.967 | 0.876 |

**Supplementary Table 6. Percent of negative interactions in microbial network constructed based on GBLM, Spearman, or Pearson correlation matrix.**

|  | **EndAECOPD** | | | **SRP066375** | | |
| --- | --- | --- | --- | --- | --- | --- |
|  | **HC**  **(n=44)** | **COPD (n=143)** | ***P* value** | **HC**  **(n=116)** | **COPD (n=74)** | ***P* value** |
| Spearman | 38.1%  (975/2556) | 20.5%  (570/2775) | **<0.0001** | 42.8%  (836/1953) | 38.9%  (760/1953) | **0.013** |
| Pearson | 55.0%  (1405/2556) | 50.0%  (1387/2775) | **<0.0001** | 53.6%  (1047/1953) | 50.3%  (983/1953) | **0.040** |
| GBLM | 33.5%  (64/191) | 24.2%  (43/178) | **0.048** | 48.3%  (69/143) | 31.6%  (48/152) | **0.003** |

**Supplementary Table 7. Demographics of the two patient clusters identified with spectral clustering in the EndAECOPD cohort.**

|  | **Cluster 1**  **(N=125)** | **Cluster 2**  **(N=18)** | ***P* value** |
| --- | --- | --- | --- |
| Age (IQR) | 65(61–71) | 66(62–70) | 0.273 |
| Male | 102(82%) | 15(83%) | 1.000 |
| BMI (kg/m^2^) | 23±3 | 22±3 | **0.035** |
| Current smoker | 17(14%) | 4(22%) | 0.305 |
| Ex-smoker | 84(67%) | 12(67%) | 1.000 |
| Pack years | 32(13–46) | 42(14–68) | 0.155 |
| FEV_1_/FVC (%) | 50(43–58) | 44(40–51) | **0.047** |
| FEV_1_ % predicted | 49(37–63) | 40(35–51) | **0.040** |
| CAT score | 11(7–15) | 9(6–13) | 0.480 |
| mMRC score | 1(1–2) | 2(2–3) | **0.007** |
| Charlson comorbidity index | 3(3–4) | 3(3–4) | 1.000 |
| Medication use |  |  |  |
| ICS+LABA+LAMA | 57(46%) | 12(67%) | 0.065 |
| ICS+LABA | 27(22%) | 4(22%) | 1.000 |
| LAMA only | 18(14%) | 0(0) | NA |

**Supplementary Table 8. Demographics of frequent and infrequent exacerbators in the EndAECOPD cohort.**

|  | **IFE**  **(N=118)** | **FE**  **(N=19)** | ***P* value** |
| --- | --- | --- | --- |
| Age (IQR) | 65(61–70) | 67(61–70) | 0.497 |
| Male | 94(80%) | 17(89%) | 0.528 |
| BMI (kg/m^2^) | 23.2±2.9 | 22.3±3.6 | 0.273 |
| Current smoker | 17(14%) | 2(11%) | 1.000 |
| Ex-smoker | 95(81%) | 17(89%) | 0.525 |
| Pack years | 32(12–44) | 36(19–54) | 0.308 |
| FEV_1_/FVC (%) | 50±10 | 48±11 | 0.359 |
| FEV_1_ % predicted | 49(38–61) | 40(33–63) | 0.306 |
| CAT score | 11(6–15) | 10(9–15) | 0.425 |
| mMRC score | 2(1–2) | 2(1–2) | **0.039** |
| Charlson comorbidity index | 3(3–4) | 3(3–4) | 0.355 |
| Medication use |  |  |  |
| ICS+LABA+LAMA | 52(44%) | 13(68%) | 0.052 |
| ICS+LABA | 27(23%) | 3(16%) | 0.765 |
| LAMA only | 15(13%) | 0(0) | NA |

IFE=infrequent exacerbators, FE=frequent exacerbators. Six COPD patients could not be connected after enrollment so that only 137 of 143 patients were classified.

**Supplementary Figure 1. Gel electrophoresis of PCR products**


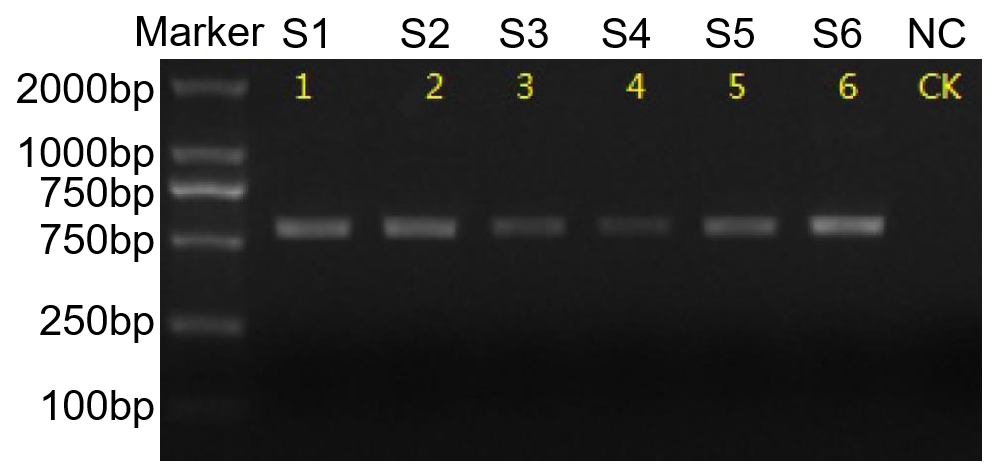


S1-6: Sample 1-6; NC: Negative control.

**Supplementary Figure 2. Validation of COPD microbial interactome disturbance using data of Haldar et al.**


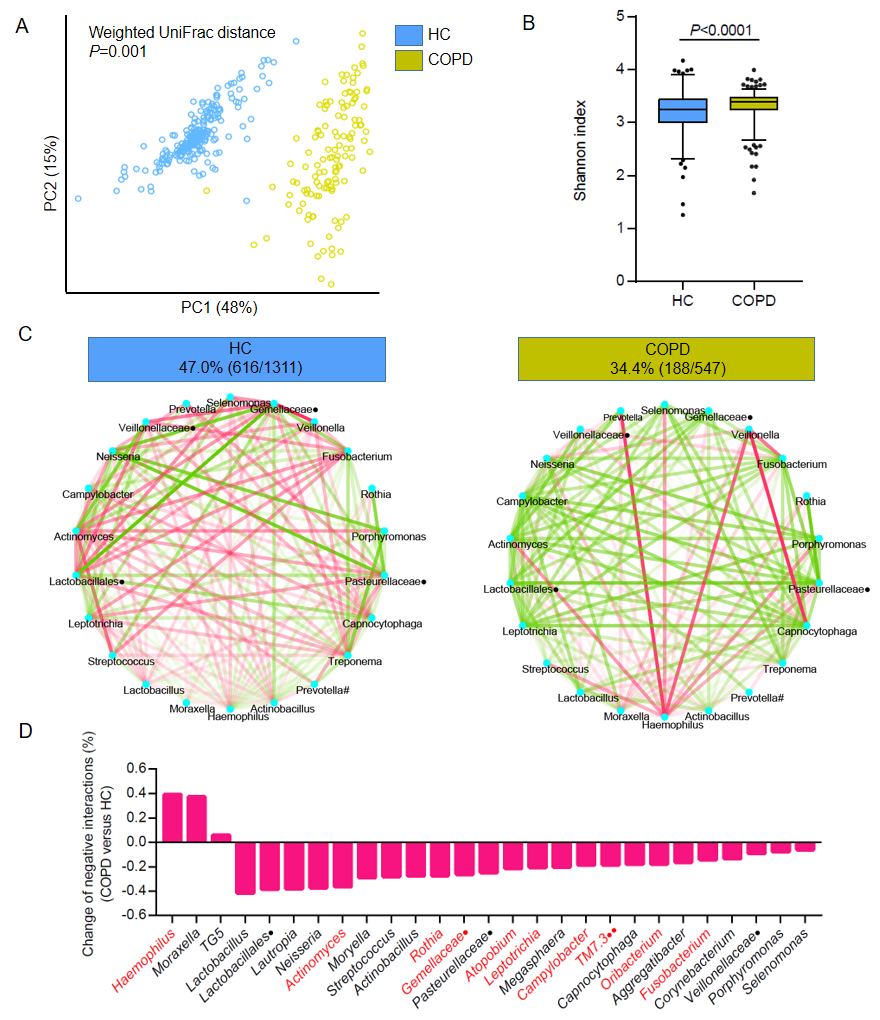


Haldar el al[13] compared COPD (N=218) versus HC (N=124) sputum microbiomes retrieved from two separate UK cohorts. Beta and Alpha diversity analysis (**A,B**) were similar to that reported in the original article. Comparison of the opposing networks between COPD and HC (**C**). Barplot showing genera with altered degree of negative interactions (percent change of >5%) between COPD versus HC (**D**). # denotes paraphyletic group; ● denotes unclassified genus; ●● denotes classified but unnamed genus.

**Supplementary Figure 3. Comparing absolute weights of negative interactions in network between COPD and HC in different cohorts**


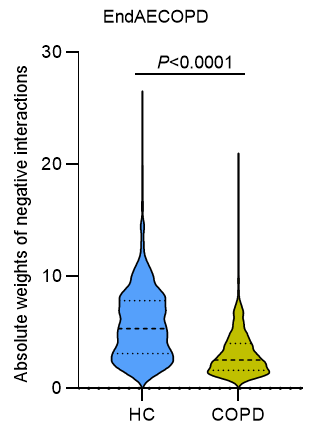

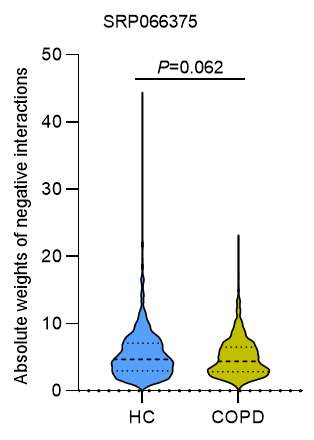

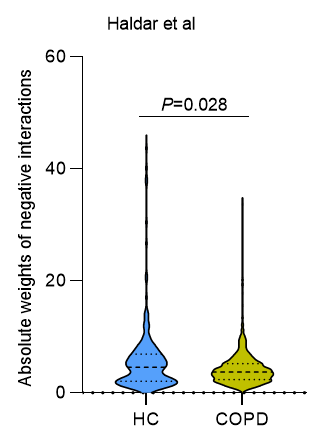


Mann–Whitney *U*-test was used to compare the absolute weights of negative interactions.

**Supplementary Figure 4. Relative abundance of *Haemophilus* and *Campylobacter* in EndAECOPD and SRP066375 cohorts.**


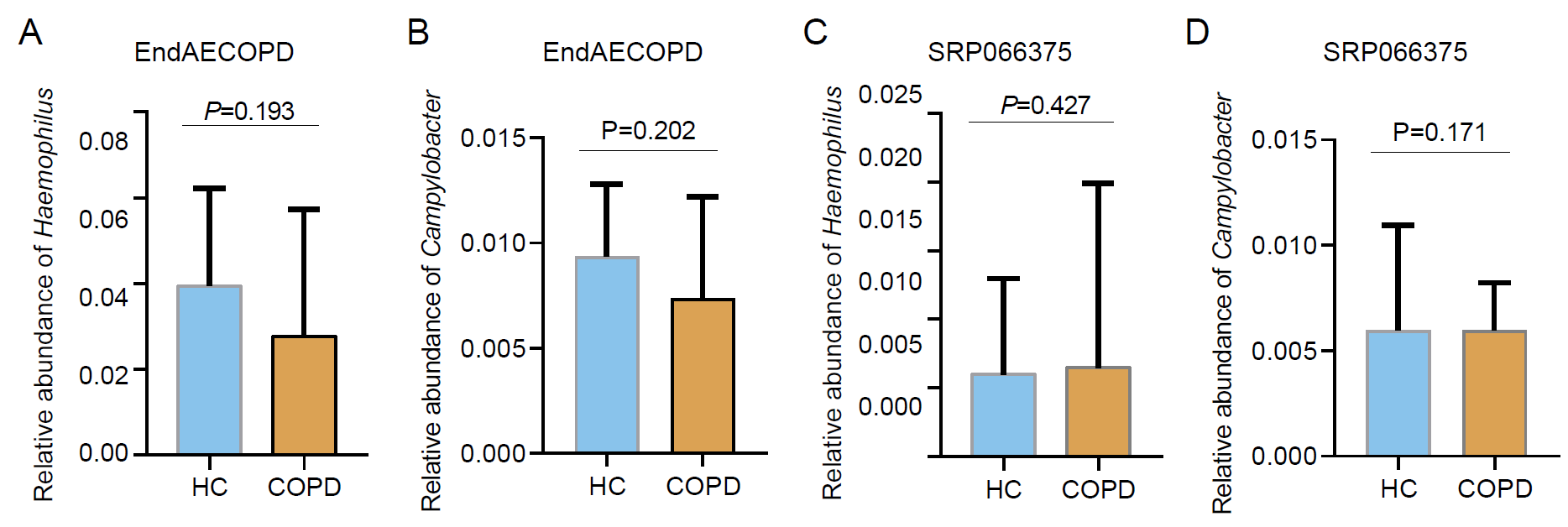


**References**

1. Hilty M, Burke C, Pedro H, et al. Disordered microbial communities in asthmatic airways. *PLoS One* 2010; 5(1): e8578.

2. Erb-Downward JR, Thompson DL, Han MK, et al. Analysis of the lung microbiome in the "healthy" smoker and in COPD. *PLoS One* 2011; 6(2): e16384.

3. Pragman AA, Kim HB, Reilly CS, et al. The lung microbiome in moderate and severe chronic obstructive pulmonary disease. *PLoS One* 2012; 7(10): e47305.

4. Sze MA, Dimitriu PA, Hayashi S, et al. The lung tissue microbiome in chronic obstructive pulmonary disease. *Am J Respir Crit Care Med* 2012; 185(10): 1073-1080.

5. Molyneaux PL, Mallia P, Cox MJ, et al. Outgrowth of the bacterial airway microbiome after rhinovirus exacerbation of chronic obstructive pulmonary disease. *Am J Respir Crit Care Med* 2013; 188(10): 1224-1231.

6. Sze MA, Dimitriu PA, Suzuki M, et al. Host Response to the Lung Microbiome in Chronic Obstructive Pulmonary Disease. *Am J Respir Crit Care Med* 2015; 192(4): 438-445.

7. Yu G, Gail MH, Consonni D, et al. Characterizing human lung tissue microbiota and its relationship to epidemiological and clinical features. *Genome Biol* 2016; 17(1): 163.

8. Einarsson GG, Comer DM, McIlreavey L, et al. Community dynamics and the lower airway microbiota in stable chronic obstructive pulmonary disease, smokers and healthy non-smokers. *Thorax* 2016; 71(9): 795-803.

9. Kim HJ, Kim YS, Kim KH, et al. The microbiome of the lung and its extracellular vesicles in nonsmokers, healthy smokers and COPD patients. *Exp Mol Med* 2017; 49(4): e316.

10. Engel M, Endesfelder D, Schloter-Hai B, et al. Influence of lung CT changes in chronic obstructive pulmonary disease (COPD) on the human lung microbiome. *PLoS One* 2017; 12(7): e0180859.

11. Diao W, Shen N, Du Y, et al. Symptom-related sputum microbiota in stable chronic obstructive pulmonary disease. *Int J Chron Obstruct Pulmon Dis* 2018; 13: 2289-2299.

12. Wang Z, Maschera B, Lea S, et al. Airway host-microbiome interactions in chronic obstructive pulmonary disease. *Respir Res* 2019; 20(1): 113.

13. Haldar K, George L, Wang Z, et al. The sputum microbiome is distinct between COPD and health, independent of smoking history. *Respir Res* 2020; 21(1): 183.

14. Ramsheh MY, Haldar K, Esteve-Codina A, et al. Lung microbiome composition and bronchial epithelial gene expression in patients with COPD versus healthy individuals: a bacterial 16S rRNA gene sequencing and host transcriptomic analysis. *Lancet Microbe* 2021; 2(7): e300-e310.

15. Millares L, Pérez-Brocal V, Ferrari R, et al. Functional Metagenomics of the Bronchial Microbiome in COPD. *PLoS One* 2015; 10(12): e0144448.

16. Wang Z, Bafadhel M, Haldar K, et al. Lung microbiome dynamics in COPD exacerbations. *Eur Respir J* 2016; 47(4): 1082-1092.

17. Garcia-Nuñez M, Marti S, Puig C, et al. Bronchial microbiome, PA biofilm-forming capacity and exacerbation in severe COPD patients colonized by P. aeruginosa. *Future Microbiol* 2017; 12: 379-392.

18. Wang Z, Singh R, Miller BE, et al. Sputum microbiome temporal variability and dysbiosis in chronic obstructive pulmonary disease exacerbations: an analysis of the COPDMAP study. *Thorax* 2018; 73(4): 331-338.

19. Mayhew D, Devos N, Lambert C, et al. Longitudinal profiling of the lung microbiome in the AERIS study demonstrates repeatability of bacterial and eosinophilic COPD exacerbations. *Thorax* 2018; 73(5): 422-430.

20. Jubinville E, Veillette M, Milot J, et al. Exacerbation induces a microbiota shift in sputa of COPD patients. *PLoS One* 2018; 13(3): e0194355.

21. Tangedal S, Nielsen R, Aanerud M, et al. Sputum microbiota and inflammation at stable state and during exacerbations in a cohort of chronic obstructive pulmonary disease (COPD) patients. *PLoS One* 2019; 14(9): e0222449.

22. O'Farrell HE, Shaw JG, Goh F, et al. Potential clinical utility of multiple target quantitative polymerase chain reaction (qPCR) array to detect microbial pathogens in patients with chronic obstructive pulmonary disease (COPD). *J Thorac Dis* 2019; 11(Suppl 17): S2254-s2265.

23. Bouquet J, Tabor DE, Silver JS, et al. Microbial burden and viral exacerbations in a longitudinal multicenter COPD cohort. *Respir Res* 2020; 21(1): 77.

24. Wang J, Chai J, Sun L, et al. The sputum microbiome associated with different sub-types of AECOPD in a Chinese cohort. *BMC Infect Dis* 2020; 20(1): 610.

25. Goolam Mahomed T, Peters RPH, Allam M, et al. Lung microbiome of stable and exacerbated COPD patients in Tshwane, South Africa. *Sci Rep* 2021; 11(1): 19758.

26. Su L, Qiao Y, Luo J, et al. Characteristics of the sputum microbiome in COPD exacerbations and correlations between clinical indices. *J Transl Med* 2022; 20(1): 76.

27. Mothur. A Multi-Center Study of the Lung Microbiome in Chronic Obstructive Pulmonary Disease. *the European Nucleotide Archive* 2015; 1: SRP066375.

28. Tangedal S, Aanerud M, Gronseth R, et al. Comparing microbiota profiles in induced and spontaneous sputum samples in COPD patients. *Respir Res* 2017; 18(1): 164.
